# Clean Label Alternatives in Meat Products

**DOI:** 10.3390/foods10071615

**Published:** 2021-07-13

**Authors:** Gonzalo Delgado-Pando, Sotirios I. Ekonomou, Alexandros C. Stratakos, Tatiana Pintado

**Affiliations:** 1 Institute of Food Science, Technology and Nutrition (CSIC), José Antonio Novais 10, 28040 Madrid, Spain; g.delgado@ictan.csic.es; 2 Centre for Research in Biosciences, Coldharbour Lane, Faculty of Health and Applied Sciences, University of the West of England, Bristol BS16 1QY, UK; Sotirios.Oikonomou@uwe.ac.uk (S.I.E.); Alexandros.Stratakos@uwe.ac.uk (A.C.S.)

**Keywords:** clean label, meat products, nitrites alternatives, phosphates alternatives

## Abstract

Food authorities have not yet provided a definition for the term “clean label”. However, food producers and consumers frequently use this terminology for food products with few and recognisable ingredients. The meat industry faces important challenges in the development of clean-label meat products, as these contain an important number of functional additives. Nitrites are an essential additive that acts as an antimicrobial and antioxidant in several meat products, making it difficult to find a clean-label alternative with all functionalities. Another important additive not complying with the clean-label requirements are phosphates. Phosphates are essential for the correct development of texture and sensory properties in several meat products. In this review, we address the potential clean-label alternatives to the most common additives in meat products, including antimicrobials, antioxidants, texturisers and colours. Some novel technologies applied for the development of clean label meat products are also covered.

## 1. Introduction

Over the last few years, food producers have identified the term “clean label” as an important market trend. Nevertheless, what does “clean label” mean? So far there, is no official nor clear definition of the term [1,2]. Asioli et al. [3] proposed two ways the consumers can interpret a product as being clean label. In a broad sense, by looking at the front of pack, consumers might assume a product is clean label if related visual claims appear, such as “free from…”, “organic”, “no additives”, etc. In a strict sense, the authors conclude that, on the back of the pack, consumers associate clean-label products with those that have a short list of ingredients, are non-synthetic, are common for the consumers, etc. Therefore, a definition of clean label should relate to the number and type of additives (synthetic or not) a product has as well as its wholesomeness. An attempt of a definition was released in the official blog of the Institute of Food Technologists: “clean label means making a product using as few ingredients as possible, and making sure those ingredients are items that consumers recognize and think of as wholesome” [4]. We believe that this is a very accurate definition of the term. It relates to all the three important aspects of the clean-label trend: short list of ingredients, trust in the ingredients and perceived healthiness. In line with this, Aschemann-Witzel et al. [5] found that consumers perceived ingredients as belonging to one of these two opposing categories: known-“natural”-good or unknown-synthetic-bad. The former being the one related with the clean-label option. It is important to remark the following finding: there is a correlation for an additive of being perceived as potentially unsafe, unhealthy or of low quality if the name is not common or difficult to pronounce [6,7]. A survey in the USA showed that, depending on the ingredient name, the perceived naturalness differs. When asked about added salt, 65.6% of the respondents considered it natural. However, when they were asked about added sodium chloride, only 32% considered it natural [8]. As with the term “clean label”, the term “natural” does not have a proper definition given by the regulators. Although consumer might understand it as a synonym of non-chemical, good and healthy, this is far from the reality where sodium chloride is the same as common salt or nitrites from synthetic origin are the same as the ones extracted from the Swiss chard. Nonetheless, consumer perception must be taken into account for product success and we do not need to forget that safety plays an essential role for the consumer, along with health, being a top-ten consumer trend in 2021 [9].

The meat industry faces important challenges, and as part of the food industry conglomerate, it needs to address changes towards clean-label options. Meat products, per definition, need to utilise an important amount of additives during their processing, so that the typical technological and organoleptic characteristics are met. In addition, many of the additives also employed during meat processing are essential to preserve the safety and shelf life of the products. Many synthetic-sounding ingredients offer functionalities that are paramount for meat quality. For this reason, nowhere else are these challenges greater than in meat production.

Additives are one of the most researched substances in the world, as they are constantly monitored by food-safety agencies, such as The European Food Safety Authority (EFSA) in the EU and (Food and Drug Administration) FDA in the USA. Within the EU, there is a list of permitted additives and their maximum level of use depending on the type of product [10]. For meat products, the list is long, including antimicrobials, antioxidants and texturisers as the most used ones, but also some other additives (such as colours, stabilisers and acidity regulators) are allowed to be used in some of the European meat products (Table 1). Consumers might perceive these additives as unhealthy or unnecessary due to their chemical-sounding name. However, all the additives used in meat processing are considered safe within the established limits by the food safety authorities. 

In this article, we present a thorough review of the clean-label options in the form of ingredients or novel technologies that can offer a real clean-label alternative to the most common additives used in meat processing. 

## 2. Clean-Label Ingredients in Meat Products

### 2.1. Antimicrobial

Consumers’ demand for safe and high-quality meat and meat products is more dynamic and diversified nowadays than in the past. They want minimally processed, easily prepared, all-natural ready-to-eat (RTE) meat products [11]. To date, the trend in consumers’ food demands, clean labelling has rapidly increased, particularly for meat products containing many food additives [12]. Researchers in parallel with producers and manufacturers have been challenged to develop healthy meat products with high quality and safety criteria. The microorganisms associated with the spoilage of meat and meat products are bacteria such as *Pseudomonas*, *Acinetobacter*, *Brochothrix thermosphacta*, *lactobacillus* spp., *Enterobacter*, as well as yeasts and moulds that can affect the organoleptic characteristics of food [13]. 

The extended use of nitrites led to growing awareness and concern about the healthiness of meat products. Numerous safety issues about nitrite have been raised because it can be converted into N-nitroso with amines in meat products, known as carcinogenic compounds to humans [14,15]. Therefore, several studies counter this challenge and help produce meat products with low or no-nitrite salts using potential alternatives with similar antimicrobial effects without causing any health hazards [16,17]. Additionally, nitrite play a major role in inhibiting the growth of foodborne pathogens such as *Listeria monocytogenes*, *salmonella* spp., *Campylobacter jejuni*, *Escherichia coli O157:H7*, *Flavobacterium*, *micrococcus* spp. and *clostridium* spp. that can cause important public health problems with million cases of foodborne diseases occurring each year [15,18].

Another additive used as preservative in meat products is sulphites. Sulphites or SO_2_ are antibacterial agents more powerful against gram-negative bacteria [19]. These additives are considered allergens as certain people have adverse reactions to their consumption, especially those sensitive to asthma, including triggering of anaphylactic reactions, hypotension, abdominal pain, dermatitis, etc. [20]. In addition to be declared as allergen content, sulphites and sulphiting agents are controlled and, in the EU, sulphites and SO_2_ are the only ones permitted at a maximum dose of 450 mg/kg and only for the following meat products: breakfast sausage, longaniza fresca, butifarra fresca and burger meat when it has 4% of cereal or vegetable. 

In the meat processing industry, several traditional thermal and novel non-thermal preservation techniques are being used to increase the products’ shelf life and enhance the sensory properties. To achieve this, meat curing is a well-developed processing stage that includes the addition of salt, nitrite and nitrate even on fresh-cut meat imparting several distinctive properties to the meat products [21,22]. The main synthetic nitrites used in the meat industry are sodium nitrite (NaNO_2_) and potassium nitrite (KNO_2_) because they are cost-effective, stable, and easy to prepare and use [23]. Before using compounds of natural origin as a replacement for nitrite, their antimicrobial efficacy should be examined, and this review provides a comparison of the published data. Foodborne pathogens can easily contaminate raw meat or meat products, and during prolonged periods of storage, spoilage microorganisms may produce an unwanted visual appearance and diminish their organoleptic properties. Research for additives of natural origin with antimicrobial activities, especially of plant origin, has notably increased in recent years [23]. Numerous natural extracts have been applied to meat and meat products, with herbs and spices being the most used as clean-label alternatives to nitrites and sulphites [24]. Among these, some plant extracts can serve as natural nitrate sources, as nitrate naturally occurs in the environment (plants, soils, water, etc.) [25]. However, nitrites of natural origin do not offer any healthier advantage towards synthetic nitrites, and they only provide a clean-label option for the consumer. Table 2 presents some potential antimicrobial alternatives from natural origin for nitrite and sulphites that can be used effectively in clean-label meat products.

Removing nitrite from meat products could be problematic because of its high antimicrobial efficacy. Hence, McDonnell et al. [33] evaluated several compounds for their antimicrobial efficacy against *L. monocytogenes* to uncured and alternative cured RTE processed meat and poultry products. The addition of vinegar, lemon and cherry powder blend (1.5%) delayed the growth of *L. monocytogenes* inoculated on the surface of cured ham and deli-style turkey breast. They suggested using the three antimicrobials on uncured roast beef as no growth of *L. monocytogenes* was observed after 12 weeks of storage at 4 °C. Moreover, *L. monocytogenes* effectively inhibited and decreased by 4 and 3 Log on RTE bologna type turkey meat coated with Nisaplin and Guardian (antimicrobial gelatin) films, respectively, after 56 days of refrigeration (4 °C) storage [34]. The efficacy of chitosan coating as an alternative to chemical protective additives demonstrated by Bostan and Mahan [35] on sausages. All sausages were dipped into 0.25, 0.50 and 1.00% chitosan solutions prepared with 1.00% acetic acid. The authors observed that the shelf life of the products increased and that 0.25% chitosan concentration was enough to inhibit the growth of aerobic bacteria, whereas higher concentrations were needed to inhibit the lactic acid bacteria (LAB). Soultos et al. [36] observed a positive effect of chitosan (0.50 and 1.00%) against the total viable count, LAB, *pseudomonas* spp., *B. thermosphacta*, *Enterobacteriaceae*, yeasts and moulds on Greek-style fresh pork sausages. Golden et al. [37] evaluated the efficacy of antimicrobial blends containing dried vinegar (DV), together with fruit and spice extracts with salt, against *C. perfringens* in uncured ham compared to traditionally cured ham. They manifested that combining the clean-label antimicrobials used had similar inhibition effects against *C. perfringens* in uncured compared to traditionally cured ham. 

Additionally, a broad range of essential oils (EOs) with antimicrobial effects is widely used on meat products to prevent the growth of foodborne pathogens and spoilage microorganisms and extend the shelf life. EOs are secondary metabolites obtained from plants [38], are composed of a complex mixture of volatile compounds of low molecular weight and are characterised by being mainly liquid at room temperature [39]. Oregano oil has been extensively used on meat with positive results against common spoilage microbiota [40,41,42] and pathogens such as S. Enteritidis [43], S. typhimurium [28,41], *S. aureus* and *L. monocytogenes* [44]. Interestingly, Hernández-Hernández et al. [45] used a novel method to encapsulate Mexican oregano (*Lippia graveolens Kunth*) EO and found that it was efficient to control the naturally occurring microbiota of fresh pork meat during cold storage. Although it is challenging to replace nitrite with a single antimicrobial compound owing to its broad-spectrum activity [46], especially against inactivation of *C. botulinum* spores in cured meat products [21], a combination of nitrite and different antimicrobial agents may be successful. In this way, De Oliveira et al. [47] reported that different levels of winter savoury with 100 ppm of sodium nitrite allowed them to control the growth of *C. perfringens* on mortadella sausages. The authors attributed the antimicrobial activity of the EOs to the presence of carvacrol, ρ-cymene, linalool and thymol. The study by Bellés et al. [48] showed that the use of carvacrol in lamb burgers could be an option as an alternative to sulphites, as it showed a delay on microbial growth. Cui et al. [49] evaluated the antimicrobial efficacy of nutmeg, sage and clove plant extracts in a model meat food. They observed a synergistic effect of the natural extracts with 10 ppm NaNO_2_ against *C. botulinum*, showing a potential combination in the control of botulism in minimally processed meat. Furthermore, Xi et al. [50] reported that lemon and lime powders and grape seed extract are less effective against *L. monocytogenes*. Still, cranberry powder together with nitrite (150 ppm) reduced the growth of *L. monocytogenes* by 2–4 Log CFU/g in cured cooked meat. Cranberry powder, long recognised as a source of natural antimicrobials, combined with nitrite (150 ppm) and grape seed extract, also offers a potential combination to inhibit *L. monocytogenes* growth in natural and organic processed meats [50]. The antimicrobial activity of the EOs is commonly attributed to the presence of the phenolic compounds [12,44,51] that can disturb the phospholipid bilayer of the cytoplasmic membrane and damage the membrane proteins leading to increased permeability of the cell membrane. However, there are several other mechanisms leading to the inactivation of the target microorganism, such as the disruption of a variety of enzyme systems [52] and destruction of genetic material [53].

The application of EOs is partially limited due to their intense aroma, which may cause adverse organoleptic effects and limited consumer’s acceptance. To overcome this problem, novel thermal and non-thermal techniques [53,54] and the use of EOs as part of the hurdle technology together with other compounds and other processing technologies, such as the encapsulation of EOs in nanostructures, are essential to improve the shelf life and the sensory attributes of meat products.

### 2.2. Antioxidants

Antioxidants are added to meat and meat products to extend their shelf life through the deactivation of free radicals, and thus slowing down the rancidity. Various factors can promote lipid oxidation in meat products. Based on their mode of action, primary antioxidants prevent lipid peroxidation by preventing a chain reaction, reacting directly with lipid radicals and converting them into relatively stable products; and secondary antioxidants act by donating a hydrogen atom (H·) and binding to catalysts such as metal ions [55,56]. The list of approved antioxidants is small within the EU but larger for the USA. The only synthetic “pure” antioxidants approved in the EU list are gallates, tert-Butylhydroquinone (TBHQ) and butylated hydroxyanisole (BHA), which are allowed for only one specific meat product: dried meat. Other additives that provide antioxidant capacity but also have other functions are nitrites, ascorbates, erythorbates and citrates. Even though the safety of synthetic antioxidants has been questioned, the safety of antioxidants of natural origin is not much different [57], as the chemical compounds are the same irrespective of their origin. However, consumers relate the word “natural” to “good”, as we mentioned before. For this reason, there has been an increase of the research and use of antioxidants of natural origin.

Antioxidants of natural origin have been identified in spices, herbs, fruits or vegetables and applied on meat and meat products primarily for their flavours and aroma. However, several natural extracts have been proven to offer the same functionality as their synthetic alternatives, with the advantage of being label-friendly and process compatible. Phenolic compounds are well known as a major group of natural antioxidants [28,58,59]. A growing list of clean-label natural extracts with antioxidant activity Generally Recognised as Safe (GRAS) by the FDA in the last years (USFDA, 2018) can be used in the meat industry. To name some of the commercially available antioxidants used throughout the meat industry, these are coffee, grape seed, green tea, oregano, sage (Greek and Spanish), lavender, lime, dill, parsley and rosemary extract between them being the most used in the meat industry [60,61]. Conversely, the EU has only approved rosemary extract as antioxidant additives for meat products [10], but the spices can be used as ingredients in the formulation following all the safety controls.

One of the most important natural antioxidants is 3,4-dihydroxyphenylethanol or hydroxytyrosol (HXT), showing interesting antioxidant characteristics and having beneficial effects on health [62]. Martinez-Zamora et al. [63] tested both natural (HXTo) and synthetic (HXTs) antioxidants on lamb meat burgers. Natural HXTo consisted of organic hydroxytyrosol (HXTo, sample 7% purity from olive tree leaves, 200 ppm) showed higher preservative activity in maintaining the nutritional value than the control synthetic HTX (HXTs, 99% purity, 200 ppm) made with sulphites. Rosemary, orange and lemon extracts were investigated in cooked Swedish-style meatballs, with the citrus extracts showing a 50% control of rancidity. The rosemary (water and oil soluble) extracts presented a complete elimination of rancidity after 12 days of storage at 8 °C [64]. In the same way, Kim et al. [65] also observed that rosemary extract had high antioxidant properties that could delay the onset of rancidity in meat fats. In this context, to explore for alternatives to synthetic additives, numerous industrial by-products of chestnuts (wood, flowers, leaves, shells, etc.) [66,67,68,69] and various fruits [32,70,71,72,73,74] have been used for their antioxidant activity on meat and meat products. The use of industrial by-products agrees with the circular economy concept [67]. It reduces the environmental impact of food processing and waste production while bringing benefits for the meat industry that avoids significant losses by protecting the meat products from oxidation, increasing their quality and shelf life. 

As we mentioned earlier, many natural extracts can negatively affect the aroma of meat products. However, there are several plants, such as spinach, radishes and celery, that contain more than 2500 mg nitrate/kg [25,75], and their extracts can be used as natural sources of nitrate in meat products. Celery has been extensively studied and used commercially because it does not affect the sensory attributes of meat products [76]. The addition of celery powder in cooked sausages significantly inhibited the quality deterioration during cold storage for four weeks [77]. Sausages containing celery powder (0.8%) showed comparable pH, thiobarbituric acid reactive substances (TBARS) and volatile basic nitrogen (VBN) values to the control samples containing sodium nitrite (0.01%). These results manifested that celery powder effectively protected sausages from quality deterioration and can be used as nitrite source from natural origin. Similarly, added celery juice powder and starter culture in emulsified sausages presented good quality characteristics without significant differences with the control samples containing sodium nitrite [78]. Nitrate obtained from plant sources can be used directly in the brine solution or the product together with a starter culture (to form nitrate into nitrite) or as a “cultured”, “prefermented” or “pre-converted” nitrate-containing plant source. The meat industry mainly applies the second method because they can control the specific natural pre-converted nitrites they use and their concentrations [76,79]. 

When evaluating natural antioxidant compounds that may prevent or retard protein and lipid oxidation, it is essential to consider the compound’s fat solubility, effective dose, optimum temperature, pH and thermal stability, as well as cost, availability and regulatory status. The meat industry has an excellent opportunity to utilise antioxidants of natural origin in their products, following the consumers’ demands for clean-label meat products.

### 2.3. Texturisers

Phosphates are the most widely used additive in processed-meat products because of their functional effects. Phosphates possess a certain antimicrobial effect and inhibit lipid oxidation, which condition the colour and the flavour of the products; but the main reason for their use is that they increase the water-holding capacity (WHC) affecting texture and sensory qualities [80]. Based on this, their replacement can lead to several technological limitations; therefore, it is essential to find alternatives that will not compromise the functions phosphates provide. Fibres, seaweeds and vegetable powders are ingredients with similar capacities to phosphates and could offer an opportunity towards clean-label meat products [80]. Phosphates are of concern for people with chronic kidney disease, as their excess in blood is associated with cardiovascular risk [81]. For the healthy individuals, even though phosphates present no concern with respect to genotoxicity or carcinogenicity and their acute oral toxicity is low, the EFSA found that the exposure was higher than the acceptable daily intake for some population groups in their re-evaluation of these additives in 2019 [82]. This is another reason for trying to find alternatives to phosphates in meat products. 

In general, strategies based on the reduction or elimination of phosphates have been studied in emulsion-type sausages (Table 3); however, they have been used in others, such as ham, bacon, delicatessen meats, breaded chicken products or injected poultry pieces [80].

Fibres present potential as functional alternatives to phosphate due to their technological advantages (high water- and fat-holding capacity, improved emulsion stability, and texture enhancement) and their positive effect on health [95]. In that sense, several rich-fibres components (whole seeds, fibre extracts, etc.) have been used to improve the texture and sensory attributes of meat products, mainly in those with reduced fat or reduced salt content [95]. However, in the development of free-phosphates meat products, the use of fibres as replacers is not so widespread. 

Chia seed presents several functional advantages but can also affect consumers’ health positively due to its high content of soluble dietary fibre [96]. In that sense, chia mucilage (formed after soaking chia seeds in water) has been used in powder and gelled form in two concentrations (2 and 4%) as sodium tripolyphosphate replacer in the development of bologna sausages [87]. New healthier products showed similar yield than controls, with both concentrations of mucilage, and in the two forms (powder and gel). Other alternative could be the use of mushrooms due to their high levels of nutrients (protein, polysaccharides, fibre and vitamins) and several biological benefits. Lyophilized and pulverized winter mushrooms were used in different concentrations (0, 0.5, 1.0, 1.5 and 2.0%) as sodium pyrophosphate (0.3%) replacer in emulsion-type sausages to evaluate their technological properties [89]. Over 1% of mushrooms powder, the exudation of fat from sausages was inhibited and an increase of pH was noted. Moreover, lipid oxidation of sausages was inhibited. However, it was observed that free-phosphates samples were softer [89] (Table 3). 

Fructo-oligosaccharides (FOSs) are soluble prebiotic fibres that have been used as an alternative clean-label ingredient to phosphates in the production of restructured chicken steaks and cooked hams [83,93]. For phosphates-free restructured steaks’ development, inulin was added in gel and powder form (4.5%). In the case of hams, FOSs were employed in different concentrations as substitutes for phosphates and dextrose, using response surface methodology. In general, the behaviour of these healthier products was similar when comparing with samples with phosphates. However, authors indicated the need to tolerate some processing compromises, such as a reduction in yield [83,93]. Other type of fibres used to avoid the use of phosphates was bamboo fibre. Its use in Bologna sausages (2.5 and 5%) resulted in being similar to others cited. Although some technological properties were conditioned with bamboo fibres, sausages maintained emulsion stability and yields [85].

By-products of the food industry that have a high fibre content could be a phosphate replacement that would allow for the industry to obtain healthier meat products while improving sustainability (many of them would otherwise go unutilised) (Table 3). Citrus fibre, a by-product of the fruit-juice industry, has been used in different concentrations (0.50, 0.75 and 1.00%) instead of tripolyphosphate with optimal results for some functional properties, such as adequate emulsion stability and yield [84]. However, authors considered that citrus-fibre levels must be assayed more critically depending on the content and type of protein present in the products. Aside from applying phosphates replacement strategies directly in the reformulation of the product, others have tried it in marinades for chicken products. Plum ingredients, dried plum powder and dried plum fibre (0.06%), and a blend of them (0.06%) were used to replace sodium tripolyphosphate in chicken breast fillets marinade [97]. A hedonic analysis and a 5-point just-about-right (JAR) demonstrated that the marinade of the blend of plum fibre and powder was not distinguishable from the control. Moreover, no differences were observed in cooking and thawing losses. Mango peel is another by-product that has been evaluated as a phosphate substitute to marinade chicken breast. Samples treated with mango peel showed similar cooking and thawing yield than those with marinate solution containing tripolyphosphate [86]. 

By-products obtained from the meat industry, such as porcine blood plasma or dehydrated beef proteins, could be used as phosphates alternatives and have been studied added directly to meat products (frankfurters) or through brines (for beef strip loins) [91,92]. The use of both meat industry by-products as phosphate replacers resulted in being positive regarding their yield; however, sensory quality was affected, as it increased animal taste and odour in frankfurters [91] and decreased tenderness in beef steaks [92]. 

Sea tangle (*Lamina japonica*) is a type of brown algae with water retention and binding ability that has been added to totally replace the sodium pyrophosphate (0.2%) in an emulsion-type sausage. Both 1.5 and 3% of sea tangle offered similar cooking loss to sausages without negative effects on sensory acceptability [88]. Natural calcium powders obtained from eggs and oyster shells were used individually or in combination as phosphate alternatives to formulate pork meat products [94]. It was observed that the combination of oyster (0.2%) and egg (0.3%) shell powder would enable the replacement of synthetic phosphate with desirable qualities in the reformulated products. 

Based on some of the ingredients mentioned, commercial alternatives to phosphates have been patented. An example that has been evaluated in marinade chicken-meat products is SavorPhos (Formtech Solutions Inc., College Station, TX, USA), a proprietary blend labelled as citrus flour, all natural flavourings and less than 2% of sodium carbonate [90]. The use of SavorPhos blend as replacer of a commercial phosphate blend, both in water and oil-based marinades, resulted in an optimal option in rotisserie chickens and chicken breasts. Similar yields were obtained with water-based marinades; however, the use of SavorPhos improved the yield with oil-based marinades. Moreover, texture values of breast were improved with the use of SavorPhos and without negatively affecting colour or sensory acceptability [90].

### 2.4. Colours 

Food colours are used to help improve the appearance of food products that could be affected by exposure to light, moisture, air and temperature variations, as well as to enhance the naturally occurring colours or give colour to otherwise colourless products. This type of additives comes from natural and synthetic origin and according to EU legislation [10] only a few are accepted and most of them limited to some dosage and specific products. From the additives of synthetic origin, only two are permitted for meat products within the EU: Allura Red AG and Ponceau 4R. The former can be applied for luncheon meat, breakfast sausages and burger meat, whereas Ponceau 4R can only be applied in three specific products: chorizo, salchichón and sobrasada. The clean-label alternatives for these colours are the food colours from natural origin, such as cochineal and carminic acids, as well as caramels, carotenes, paprika extracts or beetroot red. However, not all of these colours are permitted in the aforementioned products (Table 1). In addition, some of the food colours might present poor stability to light and time (such as beetroot red or paprika extracts), are not soluble in fat (such as cochineal) or are not soluble in water (such as carotenes) [19]. A problematic with food colours is the consumer perception of their use. Some might have a negative perception as food colours can mask other colours in the food product [98] and also for the relationship of some of them with attention-deficit/hyperactivity disorder in children [99]. Consumers might perceive a meat product as clean label if it has food colours of natural origin in it, but even these food colours can dissuade the consumer if the food colour is not a recognizable ingredient in that product, e.g., caramel in sausages. For this reason, the use of food colours in clean-label meat products should be limited to the few already accepted in the traditional recipes.

## 3. Novel Technologies for the Development of Clean-Label Meat Products

Thermal processing in addition to the use of additives, have been the only generally recognized methods for reducing food spoilage. However, the high temperatures used during these processes induce changes in the structure of food and losses of consistency and, in addition, lipid oxidation, which is the main cause of rancidity. These negative effects on the nutritional and sensory properties and the probable health risks have given rise to new technologies called non-thermal processing/mild processing/hurdle techniques [100]. High-pressure processing (HPP), ultrasound and packaging—mainly modified atmospheric packaging (MAP)—are non-thermal techniques that currently are gaining interest in the development of minimally processed food products. However, these techniques also need of an optimisation step to maintain the product quality while also extending or maintaining its shelf life.

High-pressure processing (HPP) is a treatment based on the application of high pressure (100–800 MPa), at mild temperatures (<45 °C), that is uniformly distributed through the product by a liquid transmitter. The utilization of HPP allows us to inactivate microorganisms and enzymes for a longer period without the need of chemical additives. Nonetheless, to assure food safety and to extend shelf life, the applied pressure and the temperature must be chosen according to the characteristics of the product [101]. In general, the treatment involves a minimal impact on sensory quality and nutritional value, but the noticeable differences in thermal and aggregative behaviour of proteins can condition the products’ colour and texture [102]. In beef patties, the texture and cooking loss increased with higher pressure levels [103], but a contrary effect was observed in beef gels in which HPP treatment improved the yield and texture parameters [104]. Furthermore, Maksimenko et al. [104] observed a decrease in colour values of beef gels under HHP treatment. On the other hand, as a consequence of the aggregation that HPP caused on proteins, the digestion of the meat can be improved [105]. However, high-pressure treatment may also induce lipid oxidation depending on the processing time and the pressure level applied [101]. However, this negative effect could be solved by using antioxidants of natural origin, thus maintaining the condition of clean label. For example, the use of sage powder on beef burgers pressurized at 600 MPa retarded the lipid oxidation of products over 60 days of chilled storage [106].

The introduction of the ultrasonic treatment promotes the production of pro-health, minimally processed food, which is currently very popular among consumers. Power ultrasound is a non-thermal processing technology that uses sound energy at frequencies higher than human audible range (>20 kHz) and lower than microwave frequencies (10 MHz) with many applications on muscle products, included meat tenderization, acceleration of maturation and mass transfer, and shelf-life extension [107]. Moreover, is a treatment characterized with a low impact on the organoleptic properties and the nutritional value of meat products. The use of ultrasound reduces microbial contamination due to its capacity to cause damage on biological cells, especially microbial cell membranes [108]. In addition, the use of ultrasound may allow us to reduce the use of additives, such as phosphates, due to its ability to improve the emulsification and gelling properties of proteins [109,110]. The characteristics of this technology make it attractive to reduce or even eliminate the use of additives and obtain clean-label meat products [108,111].

In addition, PEF (Pulsating Electric Field) or Pulse Light are non-thermal technologies that are receiving increased attention. Both technologies, in comparison with conventional thermal sterilization make it possible to achieve effective inactivation of microorganisms in a much shorter processing time and using less energy [108]. Moreover, the impact on nutritional and sensory characteristics of the final products is, in general, minimal.

Food packaging is an indispensable element that serves as the protection from contamination, external environment and mechanical damage. Currently, a new generation of packaging is emerging with several functions that, among others, extend the shelf life of meat products. For example, it has been observed that the combination of vacuum-packaging technology and shrinking largely extends shelf-life in comparison with traditional packaging [108]. In addition, this packaging is growing as an eco-friendly technology due to the use of biodegradable films. The new packaging materials are developed by considering not only the sustainability of their materials but also to extend shelf life, in a healthier and convenient way. The packaging that not only acts as a barrier from the outside environment but also has some active functions towards improving the shelf life is called active packaging. There are four classes of active packaging depending on the function: scavenging or absorbing, emitting, creating barriers and regulating [112]. The first class comprises mainly gas or liquid absorbers and is barely used in meat products; however, in fresh meat, they are more popular (e.g., sachets that absorb losses from fresh meat). Within the active packaging, emitting antioxidants and creating antimicrobial barriers are the most popular functions for meat products in order to prevent oxidation and microbial spoilage, and thus improving shelf life. The use of edible coatings with antioxidants and/or bioactive compounds (as the ones mentioned in Section 2.1) are being tested in different meat products. Zhao et al. [113] found that chitosan and carvacrol starch packaging films delayed microbial spoilage by up to 25 days in ham. A novel edible film made up of calcium alginate was developed by Noor et al. [114] that included *Asparagus racemosus* as bioactive ingredient. The use of this film prevented the lipid oxidation and improved the storage quality of a model meat product. A recent and thorough review of edible coatings as active packaging in meat products can be found in [115]. Consumers might perceive some risks associated with this new active packaging (technology acceptance, toxicity of new materials, economic risk, malfunction, etc.) and, thus, reject it. Although most of the attitudes towards active packaging are neutral to mildly positive, there is low familiarity with it, and if educational communication is not provided of the information of its value (i.e., extending shelf life), consumers might reject this technology [116].

## 4. Conclusions and Future Trends

The use of some additive is so extended in the manufacturing of meat products that the meat industry did not worry about finding alternatives until very recently. Consumers are demanding safe, nutritious and healthier meat products and have put the focus on the additives they contain. A clean-label meat product should only contain the ingredients from the traditional recipes easily recognised by the consumers. Some additives, such as texturisers or colours, are being replaced with alternative options. However, avoiding the use of some additives can create situations where food safety is at risk. Some alternatives rely on the origin of the additive: natural vs. synthetic (e.g., nitrites from green vegetables vs. synthetic nitrites), as natural is perceived as a good trait for most of the consumers. This would be enough for the industry, as products with “natural” alternatives will be perceived as being clean label. Nonetheless, the health problems associated with some additives do not distinguish if the substance is extracted from the nature or synthesised in a laboratory, the chemical component remains the same. We believe that future research should focus on the application of synergistic alternatives, such as a combination of novel technologies and the use of preservatives with no health implications. There is a surge in different antioxidants and antimicrobials from natural sources, but these would need to be thoroughly evaluated before being utilised as alternatives just for being “natural”. Innovations in the packaging industry are yet to be widely applied in the meat industry. Once they are fully developed, they will make an important impact on the products’ shelf life in a sustainable manner. The meat industry and meat scientists should explore further the clean-label alternatives to develop safer, nutritious and healthier meat products.

## Figures and Tables

**Table 1:** Additives permitted in the EU for meat product according to Reference [10].

E-Number	Additive Names	Max Dosage (mg/kg)	Permitted Products
E120	Cochineal, carminic acid, carmines	100	Sausages, pates, terrines, breakfast sausages (min 6% cereal) and burger meat (4% vegetables or cereal)
		200	Chorizo, salchichón
		*quantum satis*	pasturmas
E129	Allura Red AG	25	Luncheon meat, breakfast sausages (min 6% cereal) and burger meat (4% vegetables or cereal)
E124	Ponceau 4R, Cochineal Red A	250	Chorizo, salchichón
		200	Sobrasada
E150a–d	Caramels	*quantum satis*	Sausages, pates, terrines, breakfast sausages (min 6% cereal) and burger meat (4% vegetables or cereal)
E160a	Carotenes	20	Sausages, pates, terrines
E160c	Paprika extract, capsanthin, capsorubin	10	Sausages, pates, terrines
E162	Beetroot Red, betanin	*quantum satis*	Sausages, pates, terrines
E200–203	Sorbic acid-sorbates	1000	Pates, aspic
E210–213	Benzoic acid-benzoates	500	aspic
E214–219	p-hydroxybenzoates	1000	pates
E220–228	Sulphur dioxide-sulphites	450	breakfast sausages, burger meat (4% vegetables or cereal), salsicha fresca, longaniza fresca, butifarra fresca
E249–250	Nitrites	150	Non-sterilised meat products
		100	Sterilised meat products (F_0_ > 3.00)
E251–E252	Nitrates	150	Non heat treated meat products
E300–301	Ascorbic acid, sodium ascorbate	*quantum satis*	Foie gras, foie gras entier, blocs de foie gras/Libamáj, libamáj egészben, libamáj tömbben
E310–320	Gallates, TBHQ and BHA	200	Dehydrated meat
E315–316	Erythorbic acid, sodium erythorbate	500	Cured meat products and preserved meat products
E338–452	Phosphoric acid-phosphates-di-, tri- and polyphosphates	5000	Except foie gras, foie gras entier, blocs de foie gras, Libamáj, libamáj egészben, libamáj tömbben
E385	Calcium disodium ethylene diamine tetra-acetate (Calcium disodium EDTA)	250	Libamáj, libamáj egészben, libamáj tömbben
E392	Extracts of rosemary	150	Dehydrated meat, heat treated and non-heat treated meat products excluding dried sausage
		100	Dried sausage
E427	Cassia gum	1500	Heat treated meat products
E473–474	Sucrose esters of fatty acids-sucroglycerides	5000	Heat treated meat products except foie gras, foie gras entier, blocs de foie gras, Libamáj, libamáj egészben, libamáj tömbben
E481–482	Stearoyl-2-lactylates	4000	Minced and diced canned meat products
E959	Neohesperidine DC	5	As flavour enhancer only, except for foie gras, foie gras entier, blocs de foie gras, Libamáj, libamáj egészben, libamáj tömbben

**Table 2:** Studies on the application of clean-label antimicrobial compounds on meat products.

Antimicrobial	Dosage	Product	Target	Main Effects	References
Clove (*Syzygium aromaticum*) EO	5 and 10%	Ground beef	*L. monocytogenes*	*L. monocytogenes* population completely inactivated after 3 days of storage at 0, 8 and −18 °C (10% clove oil) and inhibited with 5% clove oil	[26]
Cinnamon (*Cinnamomum cassia*) EO	2.5 and 5.0%			*L. monocytogenes* counts reduced by 3.5–4.0 Log CFU/g after 7 days at 0 and 8 °C and after 60 days at −18 °C (5% cinnamon oil)	
Oregano oil and Sodium nitrite	400 pm and 50–100 ppm	Minced pork	*C. botulinum*	The synergistic effect of oregano oil and NaNO_2_ inhibited the growth of *C. botulinum*	[27]
Cinnamon EO and Grape seed extract	0.02–0.04% and 0.08–0.16% individually and in combination	Lyoner-type sausages	Lactic acid bacteria (LAB), Total viable count (TVC), Psychrotrophic count, mould and yeast count, and *C. perfringens*	Combination of cinnamon oil with grape extract 0.04 and 0.08%, respectively, reduced the final population of all counted microorganisms after 40 days, at 4 °C The combined effect of cinnamon oil with grape extract 0.04 and 0.16% reduced *C. perfringens* by 1.72 Log CFU/g at the end of storage	[24]
Grape seed extract, Pine bark extract, and Rosemary extract	1% for each extract applied separately	Ground beef	*E. coli* O157:H7, *L. monocytogenes* and *S. Typhimurium*	After 9 days of storage at 4 °C, *E. coli* O157:H7 reduced by 0.62, 0.66 and 0.18 Log CFU/g; *L. monocytogenes* by 1.01, 1.34 and 0.89 Log CFU/g; and *S. Typhimurium* by 1.11, 1.33 and 1.06 Log CFU/g, respectively, by 1% grape seed, 1% pine bark and 1% rosemary extract, compared with the control samples	[28]
*Ziziphora clinopodioides* EO and Nisin	0.1–0.2% and 250–500 IU/g individually and in combination	Raw beef patty	TVC, psychrotrophic and Enterobacteriaceae count and *Staphylococcus aureus* and *E. coli* O157:H7	All treatments affected the growth of TVC, psychrotrophic and Enterobacteriaceae count, as well as *S. aureus* and *E. coli* O157:H7 Treatment with 0.2% EO+ 500 IU/g nisin presented the highest effect on microorganisms during storage for 9 days, at 4 °C *E. coli* O157:H7 and *S. aureus* counts were under the detection limit after 7 days, at 4 °C	[29]
Nisin and Lactoferrin	0, 100 and 200 µg/g individually and in combination	Turkish style meatball	TVC, LAB, Total psychrophilic bacteria, *Pseudomonas* spp., sulfite-reducing anaerobic bacteria, yeast and mould, and coliforms, *E. coli*, Total staphylococcae count, and *S. aureus*	All groups of microorganisms significantly reduced after treatment with nisin and lactoferrin alone or in combination after 12 days of storage at 4 °C Nisin (100 μg/g) and lactoferrin (200 μg/g) reduced the coliform (> 5-Log CFU/g) and *E. coli* population to undetectable level after 3 days, at 4 °C Nisin (200 μg/g) and lactoferrin (100 μg/g) effectively reduced *S. aureus* by 3.50 Log CFU/g	[30]
Lysozyme Nisin and Disodium ethylenediaminetetraacetic acid (EDTA)	250 ppm, 250 ppm and 20 mM in combination	Ostrich Meat Patties	TVC, LAB, *Pseudomonas* spp., Enterobacteriaceae and *L. monocytogenes*	*L. monocytogenes* population decreased below the detection limit of 2.00 Log CFU/g and LAB counts reduced about 2.00 Log CFU/g after treatment on patties packaged in air and vacuum and stored at 3 °C for 8 days	[31]
Tomato, red grape, olive and pomegranate by-product extracts	1000 mg/kg	Lamb meat patties	Mesophilic bacteria, Psychrotrophic counts, LAB, Enterobacteriaceae, and *L. monocytogenes* and *Salmonella* spp.	Microbial counts on lamb patties packed in MAP and stored at 2 °C (7-day storage) after treatment with by-product extracts were significantly lower than control samples Results showed the absence of *L. monocytogenes* and *Salmonella* spp.	[32]

**Table 3:** Ingredients used as phosphates alternatives in the development of clean-label meat products.

Ingredient	Meat Product	Effects in Meat Products	Reference
Inulin (powder or gelled)	restructured chicken steaks	Maintain sensory scores.Better juiciness scores with gelled form. Oxidative and microbiological stability during frozen storage.	[83]
Citrus fibre	Cured bologna sausages	Similar emulsion stability and yield. Good behaviour during chilled storage.	[84]
Bamboo fibre	Bologna sausages	Sensorially accepted	[85]
Mango peel	Chicken marinade breast	Similar cooking/thawing yield	[86]
Chia mucilage (powder and gelled)	Bologna sausages	Reduced chewy and firm. With 2% of mucilage better emulsion stability and sensory acceptability	[87]
Sea tangle	emulsion type sausage	Similar cooking loss, overall acceptability	[88]
Winter mushroom powder	emulsion type sausage	No negative effects in colour and sensory parameters with <2%	[89]
Dried Plum Products	Chicken marinade fillets	similar sensory characteristics and yield	
SavorPhosp (commercial blend)	Rotisserie chickens and chicken breasts	Yield improved. No negative effects on technological and sensory properties	[90]
Porcine blood plasma	Frankfurter sausages	Similar water holding capacity, cooking loss and texture. Modified flavour.	[91]
Dehydrated beef protein	Beef strip loin steaks	Similar sensory characteristics, colour and microbial stability. Lower oxidation stability and tenderness.	[92]
Fructo-oligosaccharides (FOS)	Cooked hams	Higher cooking loss, satisfactory technological quality.	[93]
Calcium powders from egg and oyster	Cooked meat products	Similar yield and texture properties lighter colour.	[94]
